# An Open-Source Wireless Electrophysiology System for In Vivo Neuronal Activity Recording in the Rodent Brain: 2.0

**DOI:** 10.3390/s23249735

**Published:** 2023-12-10

**Authors:** Alexander Erofeev, Ivan Antifeev, Egor Vinokurov, Ilya Bezprozvanny, Olga Vlasova

**Affiliations:** 1 Laboratory of Molecular Neurodegeneration, Graduate School of Biomedical Systems and Technologies, Institute of Biomedical Systems and Biotechnology, Peter the Great St. Petersburg Polytechnic University, 195251 Saint Petersburg, Russia; antifeevie@bk.ru (I.A.); vinokurov.ek@edu.spbstu.ru (E.V.); bezprozvannyj@spbstu.ru (I.B.); 2 Laboratory of Methods and Instruments for Genetic and Immunoassay Analysis, Institute for Analytical Instrumentation Russian Academy of Sciences, 198095 Saint Petersburg, Russia; 3 Department of Physiology, University of Texas Southwestern Medical Center at Dallas, Dallas, TX 75390, USA

**Keywords:** neural activity, in vivo recording, open-source neurophysiology tools, monitoring brain activity, wireless electrophysiological system, microelectrode, neuroscience equipment

## Abstract

Current trends in neurobiological research focus on analyzing complex interactions within brain structures. To conduct relevant experiments, it is often essential to employ animals with unhampered mobility and utilize electrophysiological equipment capable of wirelessly transmitting data. In prior research, we introduced an open-source wireless electrophysiology system to surmount these challenges. Nonetheless, this prototype exhibited several limitations, such as a hefty weight for the wireless module, redundant system components, a diminished sampling rate, and limited battery longevity. In this study, we unveil an enhanced version of the open-source wireless electrophysiology system, tailored for in vivo monitoring of neural activity in rodent brains. This new system has been successfully tested in real-time recordings of in vivo neural activity. Consequently, our development offers researchers a cost-effective and proficient tool for studying complex brain functions.

## 1. Introduction

Modern research trends are primarily focused on the development of and improvements in methods for recording and analyzing neural activity with high spatial and temporal precision. Electrophysiological investigations in vivo represent an important tool for conducting such experiments.

Electrophysiological recording enables the comprehensive examination of neural activity, ranging from the spiking behavior of individual neurons to the fluctuations in neuronal populations, such as local field potentials. This method provides a more comprehensive understanding of cognitive, sensory, and motor functions. Furthermore, in vivo electrophysiology is employed to investigate neurodegenerative diseases, including Alzheimer’s disease [1,2], Parkinson’s disease [3], and Huntington’s disease [4]. By recording neuronal activity in animal models, researchers can observe changes in electrical patterns and identify abnormalities associated with disease development, which could provide a basis for developing potential therapeutic agents.

In vivo electrophysiology experiments necessitate specialized equipment, which includes electrode arrays [5,6,7], data acquisition systems, amplifiers, and software for data visualization and analysis. Additionally, various companies are presently prominent in the market for producing commercial electrophysiological systems tailored for in vivo experiments. These companies include Intan Technologies (Los Angeles, CA, USA), Neuralynx (Bozeman, MT, USA), Tucker-Davis Technologies (Alachua, FL, USA), Blackrock Neurotech (Salt Lake City, UT, USA), Plexon (Dallas, TX, USA), Axona (St Albans, UK), and Multichannel systems (Reutlingen, Germany).

Commercial systems offer numerous advantages and are primarily engineered with user friendliness in mind, to facilitate the easy setup and operation of the equipment. Moreover, companies that offer commercial systems often provide dedicated technical support and services. These systems are designed to seamlessly integrate with other components and accessories from the same manufacturer, ensuring compatibility and streamlining data collection. Additionally, commercial systems undergo stringent testing and quality control procedures.

However, commercial projects also come with several disadvantages, one of which is the higher cost compared to alternative open-source systems. Researchers must consider not only the initial cost but also the recurring maintenance expenses and potential charges for software upgrades. In addition, some commercial systems use proprietary hardware or software, which does not allow for customization or modification to suit specific needs. This can be a disadvantage for researchers with unique experimental requirements. All of these disadvantages severely limit the availability and dissemination of in vivo electrophysiological applications.

Considering all these factors, the development of open-source in vivo electrophysiological systems is particularly relevant at present. Due to the prevalence of free and low-cost software that makes electrophysiology systems available to researchers working under financial constraints, these systems are cost-effective compared to commercially available options. In addition, these systems are very flexible, allowing them to be customized to meet specific research objectives. Open-source projects also have active user communities that promote transparency, collaboration, and the sharing of ideas and code.

Currently, several examples of open-source systems in the field of electrophysiology exist: an open-source, wireless electrophysiology system [8]; OSERR—an open-source standalone electrophysiology recording system for rodents [9]; Open Ephys (https://open-ephys.org/ (accessed on 1 September 2023)), which offers hardware and software for data collection and analysis; SpikeGadgets (https://spikegadgets.com/ (accessed on 1 September 2023))—a software package for electrophysiology with the ability to sort spikes in real time; and Kilosort (https://www.ontologic.ly/tools/kilosort (accessed on 1 September 2023))—a spike sorting algorithm. Additionally, a prior study conducted by our laboratory’s team resulted in the development of an open-source wireless electrophysiology system for recording neuronal activity in rodents [10]. The results of this research include the development of a rechargeable wireless wearable module with a battery life of approximately 3 h, a base station responsible for receiving and transmitting data from the wireless module, and software for visualization and data recording.

However, the initial version of the wireless electrophysiology system (WES) [10] exhibited a notable drawback: an excessive number of components, leading to manufacturing complexities; a hefty weight for the wireless module, a diminished sampling rate, and limited battery longevity. Consequently, in response to these issues, we designed an enhanced and simplified version of the WES while maintaining its functionality, called the second version.

## 2. Materials and Methods

The following sections of this paper describe the components of a wireless electrophysiology system. The term ‘electrophysiology system’ encompasses a wireless wearable module, an external PC equipped with user software, and a Bluetooth data transceiver. Section 2.1 describes the fabrication process of the 12-channel microelectrode utilized in this study. Section 2.2, Section 2.3 and Section 2.4 provide detailed insights into the individual components of the electrophysiology system. Section 2.5, Section 2.6 and Section 2.7 expound upon the methodologies and equipment employed for microelectrode implantation and in vivo recording of neuronal activity in the laboratory mice brain.

### 2.1. Microelectrode

**Figure 1:**
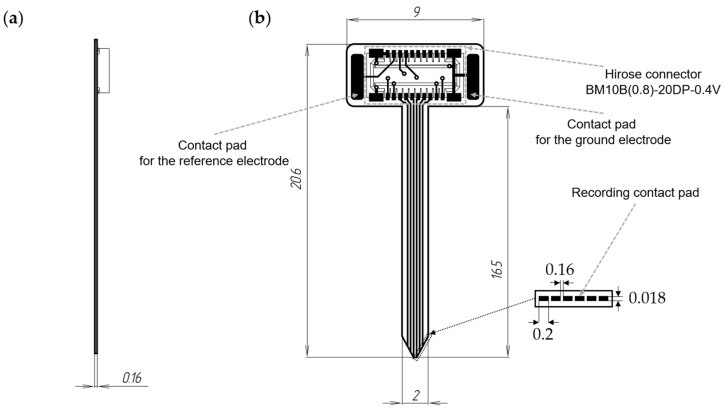
Schematic representation of a 12-channel microelectrode: (**a**)—lateral view, (**b**)—frontal view.

To assess the functionality of the developed wireless electrophysiology system, a custom 12-channel (6 sites on each side) microelectrode (Figure 1) was designed in Altium Designer (Altium, San Diego, CA, USA). The fabrication of the microelectrode was carried out by LLC Rezonit (Moscow, Russia), a company specializing in the production and assembly of printed circuit boards. The microelectrode was constructed using a three-layer design, which included polyimide film as the substrate, copper tracks for conductivity, and polyimide film with cutouts for recording sites. The copper tracks were created by fixing copper foil onto the polyimide film and then subjecting it to photoresist and etching processes.

The microelectrode possessed a thickness of 0.16 mm, with recording sites sized at 0.2 × 0.018 mm and an inter-site distance of 0.16 mm. The total length of the microelectrode was 20.6 mm, with the lead length, including the recording part, measuring 16.5 mm. To connect to the wireless module, a Hirose connector BM10B(0.8)-20DP-0.4V (Hirose Electric, Kanagawa, Japan) was utilized on the microelectrode.

After fabrication, the recording sites were coated with a 5 µm thick layer of gold. This coating was achieved by immersing the microelectrode in a 0.8 g/L gold solution and applying a current density of 15 mA/mm^2^ at 25 degrees Celsius using a laboratory power supply (AKIP, Izhevsk, Russia).

For impedance evaluation, the fabricated microelectrode was submerged in a 0.1 M phosphate-buffered saline, and an RLC meter (AMM-3035, AKTACOM, Moscow, Russia) was used to measure impedance. AC sinusoidal signals with a voltage amplitude of 5 mV and frequencies ranging from 0.1 to 1000 Hz were applied for impedance measurement. In the frequency range of 10 to 300 Hz, the impedance value ranged from 4 to 5 MOhm.

### 2.2. Wireless Wearable Module

**Figure A1:**
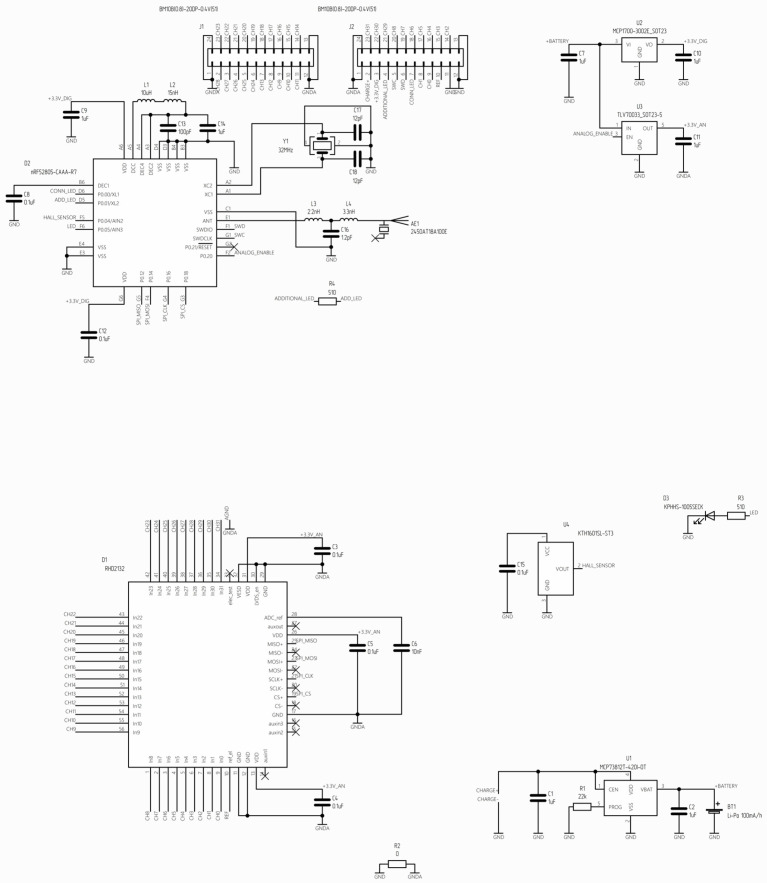
The circuit diagram illustrates the main components of the wireless module.

**Figure A2:**
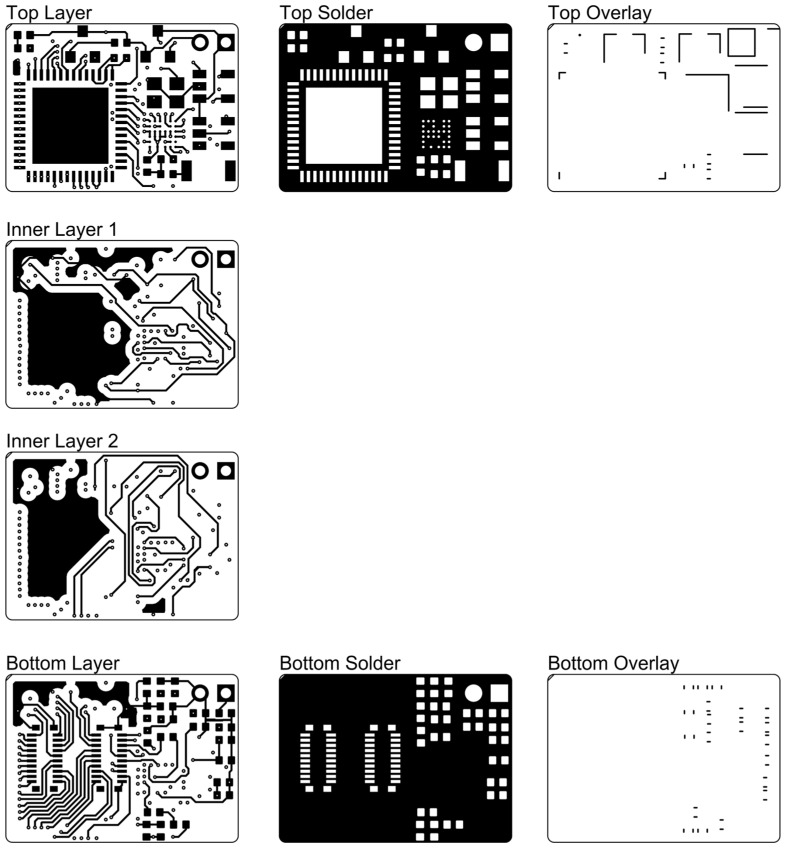
The PCB layout of the wireless module.

The printed circuit board (PCB) layout for the wireless module was created using the open-source software KiCad EDA version 7.0.6 and was subsequently sent for production. The manufacturing process, involving the fabrication of printed circuit boards and the installation of components onto the PCBs of the wireless modules, was carried out by Rezonit LLC (Moscow, Russia). The circuit diagram and PCB layout of the wireless module are shown in Appendix A (Figure A1 and Figure A2).

The list of required components, required files, and software can be found in the GitHub repository at the link—https://github.com/lmn-projects/WES-2.0 (accessed on 1 September 2023).

### 2.3. Firmware

Firmware of the wireless module was written in C language using the Keil µVision 5 utility (Keil Elektronik GmbH, Grasbrunn, Germany). The wireless module was flashed using the J-Flash Lite v7.80c utility (Segger Microcontroller, Monheim am Rhein, Germany). Detailed instructions for the firmware flashing procedure can be found in Appendix B. For the firmware flashing, the primary Hirose connector, located in the center of the wireless module, was utilized via the serial wire data (SWD) and serial wire clock (SWC) pins.

### 2.4. Software

The software responsible for operating the WES (ble_mouse.py), designed to record neural activity, was developed using the Python 3.7 programming language. The execution of this software was initiated via the command line. Instructions for using the ‘ble_mouse.py’ script can be found in Appendix B.

### 2.5. Animals

B6SJLF1 mice (Jackson Laboratory, Bar Harbor, ME, USA; strain #100012-JAX) were obtained from the Jackson Laboratory and used for microelectrode implantation. These mice were established and maintained in a vivarium with four to five mice per cage and a 12 h light/dark cycle in the animal facility. Food and water were available ad libitum. All procedures adhered to the principles of the European Convention and the Declaration of Helsinki regarding the humane treatment of animals and were approved by the Bioethics Committee of Peter the Great St. Petersburg Polytechnic University in St. Petersburg, Russia (ethical permit No. 6 from 22 August 2022).

### 2.6. Microelectrode Implantation

In order to evaluate the wireless electrophysiological system, we surgically implanted a custom-designed 12-channel microelectrode into the hippocampus of a 6-month-old B6SJLF1 wild-type mouse, utilizing a stereotaxic setup. Prior to the microelectrode implantation, the animal was anesthetized with isoflurane (1.5–2%). Following the confirmation of the absence of sensitivity, the animal’s fur was shaved in the cranial region, the skin was incised, and the skull was thoroughly irrigated with hydrogen peroxide and phosphate-buffered saline. The stereotaxic setup was employed to determine the implantation site for the microelectrode, specified in relation to the bregma coordinates as follows: anteroposterior (AP): −0.23 mm, dorsoventral (DV): −0.15 mm, and mediolateral (ML): −0.18 mm. On the opposite side of the implantation site, a small aperture was created in the skull using a dental drill to facilitate the installation of a microscrew, to which a silver wire was soldered. This wire was subsequently connected to the microelectrode’s reference contact pad.

In reference to the point determined using a stereotaxic setup, several guiding holes were created to perform a rectangular craniotomy measuring 2 × 1 mm. Subsequently, the cranial fragment and dura mater were carefully removed. In the case of bleeding, the implantation area was additionally rinsed with phosphate-buffered saline and filled with a hemostatic sponge. A microelectrode was attached to the stereotaxic apparatus using a special mount and lowered to 1.35–1.4 mm below the upper part of the skull into a pre-formed implantation area. The implantation speed of the microelectrode was set at 1 mm in 3–5 min. The external surface of the microelectrode was secured to the skull using cyanoacrylate glue until completely dry. Subsequently, the microelectrode holder was removed, and the skull was covered with light-cured dental cement. After the operation, 0.1 mL of dexamethasone was subcutaneously administered. Then, the isoflurane level was set to 0%, and the animal was transferred from the stereotaxic setup to a cage with a thermoregulating electric heating pad. In the initial days of the postoperative period, the animal was fed with soft food, such as granulated porridge.

Three weeks post-implantation, neural activity was recorded using a wireless electrophysiology system. Prior to the electrophysiological recording, a silver wire was connected to the ground contact pad of the microelectrode, after which the wire was subcutaneously introduced into the animal under anesthesia.

To address the challenges posed by parasitic vibrations, we secured a portion of the microelectrode loop to the animal’s skull using dental cement. Subsequently, the wireless module, upon connection to the microelectrode, was affixed to the skull using specialized 3M foam designed for its vibration-damping properties.

### 2.7. Computing Setup

In vivo recordings were conducted on a PC equipped with an Intel Xeon E3-1246 v3 CPU running at 3.5 GHz, 32GB DDR3 RAM, Windows 10 (Version 10.0.17763 Build 17763), and Bluetooth 5.3. The software tools employed included python 3.7.

## 3. Results

Our primary objective was to streamline the design of the initial version of the wireless electrophysiology system while retaining its existing features. This decision stemmed from numerous inquiries following the publication of our previous article [10]. The initial version of WES comprised a base charging station and a wireless wearable module, which could be paired with a 32-channel microelectrode.

In the initial version of the wireless wearable module, we utilized a 2.4 GHz wireless channel. The module incorporated the nRF24L01P (Nordic Semiconductor, Trondheim, Norway) digital transmitter, featuring 126 frequency channels and GFSK modulation, along with the STM32G071GBU6 (STMicroelectronics, Geneva, Switzerland) as a microcontroller. Additionally, the module incorporated a 32-channel amplifier chip RHD2132 (Intan Technologies, Los Angeles, CA, USA), connectors for electrodes A79027-001 (Omnetics, Minneapolis, MN, USA), optical probes A79607-001 (Omnetics, Minneapolis, MN, USA), and a 30 mAh 3.7 V lithium-ion battery LP301012 (Akyga battery, Wroclaw, Poland). These elements were assembled on a flexible four-layer PCB.

In the base charging station of the WES 1.0, we employed the STM32F746VET6 (STMicroelectronics, Geneva, Switzerland) microcontroller to control radio components, USB, and Ethernet functionalities. The microcontroller also managed the interaction protocol between wireless modules and a PC. For communication with the PC, we utilized the USB type-C interface, featuring power contacts and operating within the USB 2.0 standard bandwidth. Data transmission was handled by the nRF24L01 (Nordic Semiconductor, Trondheim, Norway) radio module, enabling simultaneous signal transmission from three wireless modules. To charge modules, we utilized the TP4054 (Youtai Semiconductor, Shenzhen, China) Li-ion battery charge controller. A custom plastic mount, equipped with specialized spring-loaded pogo pin contacts, facilitated easy fixation and reliable charging. The charging process was regulated by an optical switch ITR-9909 (Everlight, Hong Kong, China) that distinguished active modules from inactive ones.

In the WES 2.0, we simplified the system by retaining only the wireless wearable module.

### 3.1. Base Station

**Figure 2:**
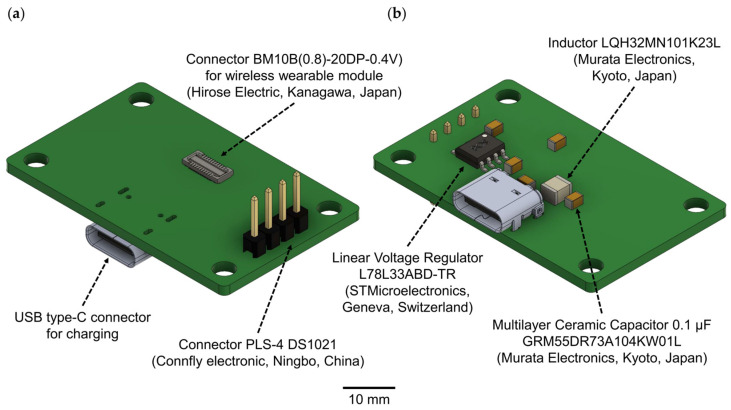
A 3D model of the charging station for the wireless wearable module in the WES 2.0: (**a**) top view, (**b**) bottom view.

In the second version of the WES, we have entirely eliminated the base charging station. Instead, a direct connection to a personal computer is established using Bluetooth protocol version 5.4 or earlier. The wireless wearable module can be charged separately, either with the provided charging station (Figure 2, Table 1) or, alternatively, it can be charged using a universal battery charger.

The USB type-C connector is used to connect the charging station and should be plugged into the USB port of a personal computer. For the connection between the wireless wearable module and the charging station, the BM10B(0.8)-20DP-0.4V connector is employed. To link to a PC for subsequent debugging and programming of the wireless wearable module, the PLS-4 connector is used in conjunction with the SEGGER J-Links programmer (https://www.segger.com/products/debug-probes/j-link/ (accessed on 1 September 2023)).

Alternatively, for debugging purposes, we recommend using the nRF52840 USB dongle (Nordic Semiconductor, Norway). This dongle is a readily available and cost-effective solution that supports a wide array of short-range wireless standards, including Bluetooth 5.4 with Bluetooth low energy, Bluetooth mesh, Thread, Zigbee, 802.15.4, ANT, and proprietary 2.4 GHz protocols. Moreover, it is compatible with macOS, Linux, and Windows 7 (and higher) operating systems. The nRF52840 USB dongle is also well-suited for use with the nRF5-SDK development environment and nRF-Connect/nRF5x software v4.3.0, both provided by the manufacturer.

### 3.2. Wireless Wearable Module

**Figure 3:**
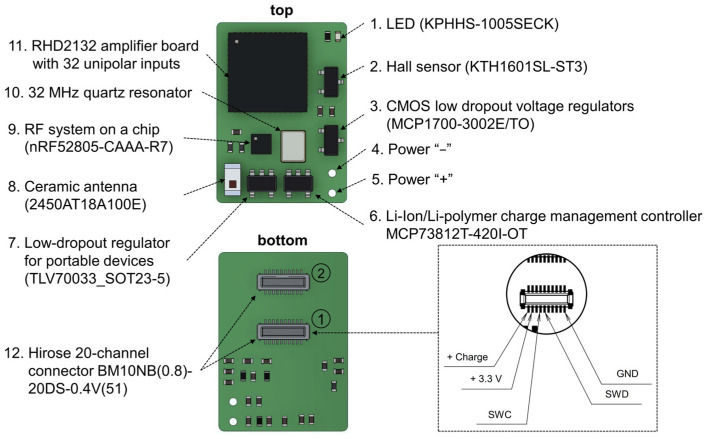
Key components of the wireless wearable module in the WES 2.0. The pinout configuration of the primary Hirose connector for the wireless module is displayed within the dotted line area. The connectors for microelectrode connection are denoted by numbers 1 and 2. The primary connector is identified as number 1.

The wireless wearable module in WES 2.0 has been designed as a single PCB, distinguishing it from the first version that used a flexible PCB consisting of two parts. The dimensions of the second version of the wireless module (Figure 3) are 18 × 13 × 3 mm, with a weight of 0.95 g without the battery and 3.19 grams when equipped with the LP601120 (Robiton, Moscow, Russia) 100 mAh 3.7 V lithium-ion battery.

The wireless wearable module is based on the NRF52805-CAAA-R7 (Nordic Semiconductor, Trondheim, Norway) receiver–transmitter microprocessor and the RHD2132 32-channel unipolar input amplifier chip (Intan Technologies, Los Angeles, CA, USA). The RHD2132 chip enables the in vivo recording of weak electrical signals by directly converting them into a digital data stream, replacing traditional analog instrumentation and digitization circuitry found in typical electrophysiological recording systems. The differential gain exhibited by the RHD2132 chip is 192 V/V.

Also, the main components of the wearable module include: two 20 position receptacle connectors, BM10NB(0.8)-20DS-0.4V(51) (Hirose Electric, Kanagawa, Japan); a ceramic antenna, 2450AT18A100E (Johanson Technology, Camarillo, CA, USA); a lithium-ion and lithium-polymer battery charge controller, MCP73812T-420I/OT (Microchip Technology Inc., Chandler, AZ, USA); a low voltage drop fixed stabilizer, MCP1700-3002E/TO (Microchip Technology Inc., Chandler, AZ, USA); a voltage regulator, TLV70033_SOT23-5 (Texas Instruments, Dallas, TX, USA); a Hall sensor, KTH1601SL-ST3 (CONNTEK Microelectronics Technology, Quanzhou, China); an LED, KPHHS-1005SECK (Kingbright, Shanghai, China); and a 32 MHz quartz resonator, NX2520SA (NDK, Tokyo, Japan).

One of the primary distinctions between the second version of the wireless module and the first iteration is the shift from the proprietary 2.4 GHz data protocol to the Bluetooth low energy protocol (Table 2). Additionally, the second version incorporates the NRF52805-CAAA-R7 (Nordic Semiconductor, Trondheim, Norway) microprocessor-receiver–transmitter. This marks a departure from the first version, which used both the 2.4 GHz RF System-on-Chip nRF24L01P (Nordic Semiconductor, Trondheim, Norway) and the microcontroller STM32G071GBU6 (STMicroelectronics, Geneva, Switzerland). The microprocessors in both systems demonstrate comparable performance characteristics (Table 3). Consequently, the second version consolidates receiving, transmitting, and processing data into a single chip, resulting in potential advantages concerning manufacturing costs and the power consumption of the wireless module. At an operational voltage of 3.3 V, the current consumption rates are 2.5 mA in standby mode and 2.6 mA in data transfer mode.

By transitioning to the NRF52805-CAAA-R7 and streamlining the entire system, we reduced the weight of the wireless module from 1.79 to 0.95 grams (Table 2). This reduction facilitated the incorporation of higher-capacity batteries, thereby substantially prolonging the operational lifespan of the module on a single charge. For instance, with a 100 mAh battery, the projected autonomous operating duration of the wireless module exceeds 35 h, given a power consumption rate of 2.6 mA.

Another notable distinction from the initial version is the capability to select the sampling rate. In WES 1.0, data were recorded at a fixed sampling rate of 300 Hz. Conversely, in WES 2.0, users have the flexibility to choose the sampling rate within a range spanning from 62 Hz to 1000 Hz (further details are provided in Section 3.3).

In addition to the changes in microchips, the previous 36-channel connector A79027-001 (Omnetics, Minneapolis, MN, USA) has been replaced with two 20-channel connectors, BM10NB(0.8)-20DS-0.4V(51) from Hirose Electric, Kyoto, Japan. The primary connector (Figure 3, first connector, located in the middle of the wireless module board) includes pins for various functionalities, including firmware flashing (SWD, SWC, +3.3V_DIG), charging (Charge +, ground (GND)), and providing power to the LED when an optrode is connected (ADDITIONAL_LED, Figure A1). The remaining pins are dedicated to the direct recording of neuronal activity, using a microelectrode implanted in the brain of a laboratory animal. The decision to replace the Omnetics connector with the Hirose connectors was made due to its greater cost and size.

Similar to the first version, the wireless module is initiated using a Hall sensor. Upon connecting the battery to the wireless module, the nRF52805-CAAA-R7 chip is activated and transitions to minimal power consumption mode. To fully activate the module, a magnet needs to be brought close to the area of the Hall sensor on the board (Figure 3) for a few seconds. Subsequently, firmware initialization occurs, and the module enters its operational state. For proper functionality and to prevent erroneous operation, the module should only be turned on after connecting it to the microelectrode connector. In the current firmware version, the wireless module remains active until the battery is either completely discharged or disconnected.

### 3.3. Software

The software for the second version of the wireless electrophysiology system is executed through the command line. To initiate its operation, the ‘ble_mouse.py’ file should be executed with specific parameters, including the desired sampling rate and the list of channels for recording. For example, to initiate the registration of the 7 and 25 channels with a sampling rate of 1000 Hz, the following command can be used: ‘ble_mouse.py 1000 7 25’.

In the current software version, the number of registration channels is limited by the selected sampling rate: two channels at 1000 Hz, four channels at 500 Hz, eight channels at 250 Hz, sixteen channels at 125 Hz, and thirty-two channels at 62 Hz. To terminate the registration process, the space bar should be pressed and held for a few seconds. As a result, a file named ‘mouse_ble_current-date_current-time’, in a .csv format, will be saved. The first column of this file will contain the number of Bluetooth protocol data packets, and the subsequent columns will contain the values of the received samples, where one sample corresponds to 5 µV.

It is important to note that the wireless module currently lacks filtering capabilities. In future iterations of the software, alongside the integration of a graphical interface, there are plans to incorporate real-time data filtering functionality.

### 3.4. Recording

**Figure 4:**
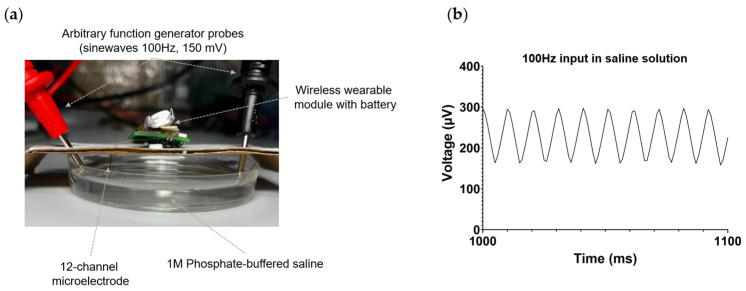
In vitro testing of a wireless electrophysiology system in PBS. (**a**) Photograph of the setup for in vitro experimental testing. (**b**) The waveform of the recorded 100 Hz, 150 mV signal injected into the saline by the wireless electrophysiology system, utilizing a 12-channel microelectrode.

The performance of the wireless electrophysiological system was initially assessed in vitro using phosphate-buffered saline (PBS). To achieve this, a 12-channel microelectrode connected to the wireless module was immersed in PBS, as depicted in Figure 4a. A titanium wire was soldered to the reference and ground pads of the microelectrode and also submerged in PBS in the following configuration: the reference electrode was positioned adjacent to the microelectrode, while the ground electrode was situated on the opposite side of the microelectrode. Employing a Tektronix AFG3022C random pulse generator (Tektronix, Beaverton, OR, USA), a sinusoidal signal with a frequency of 100 Hz and an amplitude of 150 mV was applied to the PBS. The integrity of the signal was confirmed using a Tektronix MSO2024B mixed-signal oscilloscope (Tektronix, Beaverton, OR, USA). Figure 4b illustrates the successful registration of signals in PBS by the wireless module.

**Figure 5:**
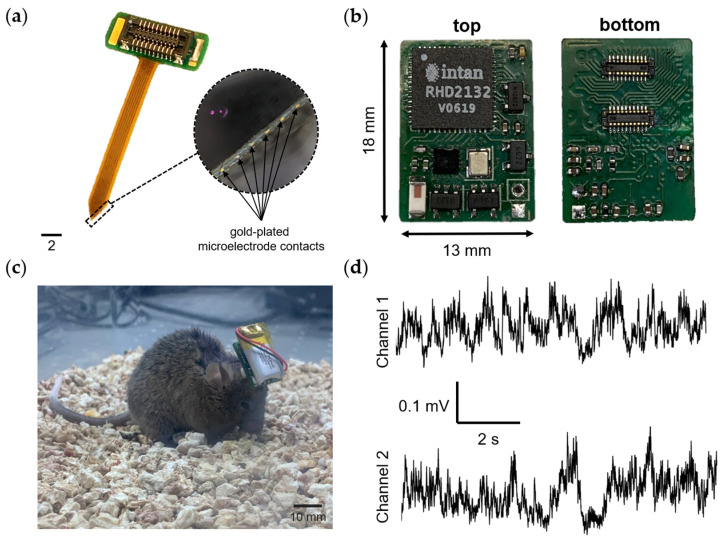
In vivo recording neural activity in the mouse brain. (**a**) Photograph of a 12-channel microelectrode. The circle highlighted by the dotted line shows the contact pads of the microelectrode for recording neuronal activity (magnification 4×). (**b**) Photo of the wireless wearable module version of the WES 2.0. (**c**) A laboratory mouse with an implanted 12-channel microelectrode, along with a connected wireless module. (**d**) An example of raw recorded neural activity from two channels, with a sampling rate of 1000 Hz.

To evaluate the performance of the wireless module in vivo, neural activity recording was conducted on a mouse. To achieve this, a 12-channel polyimide-based microelectrode (Figure 5a) was manufactured and surgically implanted into the mouse brain. The in vivo recording took place within a Faraday cage to minimize interference and noise. Two channels were recorded at a sampling rate of 1000 Hz. The obtained results are presented in Figure 5.

**Figure 6:**
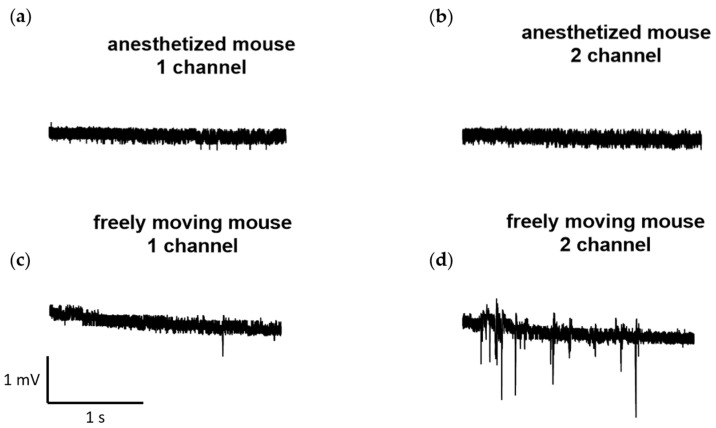
Examples of recordings obtained at a sampling rate of 1000 Hz from the developed wireless electrophysiological system. The recordings were obtained both during the state: (**a**,**b**) when the animal was anesthetized with isoflurane (1.5–2%) and (**c**,**d**) in free movement.

Furthermore, we conducted several recordings of neural activity obtained at a sampling rate of 1000 Hz using the developed wireless system for the same laboratory mouse and recording channels. These recordings were made both in a state when the animal was anesthetized with isoflurane (1.5–2%), and during free movement. The examples of the recordings are presented in Figure 6.

## 4. Discussion

In the second version of the wireless electrophysiology system, we managed to simplify the entire system by eliminating the base charging station and switching to the Bluetooth low energy protocol. Despite these simplifications, we have managed to preserve crucial characteristics, such as the number of recording channels and the ability to connect an optrode. Furthermore, we successfully decreased the weight of the wireless wearable module, enhanced its battery longevity, and increased the sampling rate.

Table 4 presents a comparative analysis of our system alongside several recently published electrophysiology systems designed for freely moving rodents. Our system distinguishes itself from its counterparts primarily through its extended battery life and the optimal combination of parameters, including the number of recording channels, as well as the size and weight of the wireless wearable device.

In addition, our system is distinguished by its comprehensive accessibility, encompassing everything from the component list to the firmware files, along with the corresponding instructions. The system predominantly employs commercially available components, except for the printed circuit board, thereby facilitating ease of replication. We believe this characteristic notably enhances its accessibility within the scientific community.

Nonetheless, the current version of the WES does have some limitations. Specifically, the use of the second BM10NB(0.8)-20DS-0.4V(51) connector (Figure 3) for the wireless module is not possible without the simultaneous use of the first connector. This limitation arises because the contacts for the ground and reference electrodes are positioned on the first Hirose connector. This issue could potentially be resolved by incorporating the necessary contacts into the second connector. However, this would reduce the total number of available contacts for recording. Alternatively, Omnetics connectors with 36 or more channels could be used, but this might increase the final size and weight of the wireless wearable module.

Another potential drawback is the sensitivity to the polarity of the connected battery wires. If the battery is connected with reversed polarity, it may damage the components of the wireless module. One approach to address this issue is the utilization of a field-effect transistor, although its size might adversely affect the overall mass and dimensional characteristics of the wireless wearable module. Additionally, it is worth mentioning that, in the current configuration, the battery is soldered to the wireless module board, which could create challenges when attempting to swiftly replace the battery for swapping or charging using a universal charger. A viable solution to this problem could involve incorporating a quick-release connector on the wireless module board and battery wires.

In the current version of the wireless module firmware, upon battery connection, the module consistently remains in the minimum power consumption mode until the Hall sensor is activated. Incomplete disconnection of the battery ultimately has a detrimental effect on the autonomy of the wireless module. The solution to this issue involves modifying the on/off algorithm as follows: in the off state, no power will be supplied to the module components, and the nRF52805-CAAA-R7 chip will remain inactive. Upon activating the Hall sensor by holding the magnet nearby for a few seconds, the module will be powered up, the firmware will be initialized, and the module will enter the operating state. Moreover, in the current firmware version, the wireless module is disabled only when the battery is either completely discharged or disconnected. This problem can be programmatically resolved by implementing a specific command in the software, which will cut off the power supply and switch the module to a disabled state.

Furthermore, although there have been advancements in the sampling rate, this parameter remains significantly inferior to that of other electrophysiological systems, as detailed in Table 4. Presently, the limitation in the number of channels constrains the selection of higher sampling rates from the available options.

In forthcoming iterations of the WES 2.0, we plan to make modifications to the on/off algorithm of the wireless wearable module, with the aim of enhancing its energy efficiency and prolonging the battery life on a single charge. Additionally, we plan to increase the sampling rate to 30 kHz when all 32 channels are operating simultaneously. Moreover, our intentions include enhancing the software by incorporating real-time visualization of recorded signals and motion correction algorithm. These improvements are anticipated to further enhance the overall performance and capabilities of the wireless wearable module.

## 5. Conclusions

The present study describes the successful development of an enhanced and simplified open-source wireless electrophysiology system for in vivo neuronal activity recording in the rodent brain. This system offers an affordable and accessible solution for researchers, fostering innovation and collaboration in neuroscience research. Future work will focus on further enhancing the system’s performance and expanding its capabilities, ultimately contributing to a better understanding of the complexities of the brain and its functioning.

## Figures and Tables

**Table 1:** Components of the charging station for the wireless wearable module.

Name	Manufacturer	Quantity	Description
BM10B(0.8)-20DP-0.4V	Hirose Electric, Kanagawa, Japan	1	20-pin connector
LQH32MN101K23L	Murata Electronics, Kyoto, Japan	1	SMD RF inductor 100 UH 10%
GRM55DR73A104KW01L	Murata Electronics, Kyoto, Japan	5	SMD multilayer ceramic capacitors 0.1 µF 1 kVDC 10% 2220 X7R
PLS-4 (DS1021)	Connfly Electronic, Ningbo, China	1	PLS connector, plug to board, single-row straight 4-pin (1 × 4), pitch 2.54 mm
L78L33ABD-TR	STMicroelectronics, Geneva, Switzerland	1	Linear Voltage Regulators 3.3 V 0.1 A Positive
USB-C31-S-RA-EH2.0B-BK-T/R	Adam Tech, Union, NJ, USA	1	USB type-C connector

**Table 2:** The key differences between the WES 1.0 and 2.0 wireless modules.

Components	WES 1.0	WES 2.0
Receiver–transmitter	nRF24L01P (ANT and 2.4 GHz proprietary protocols)	NRF52805-CAAA-R7 (Bluetooth 5.4, Bluetooth low energy, 2.4 GHz proprietary protocols)
Microcontroller	STM32G071GBU6	
Microelectrode connector	A79027-001 (36 position receptacle connector)	BM10NB(0.8)-20DS-0.4V(51) (Two 20 position receptacle connectors)
Amplifier chip	RHD2132 (32-channel amplifier)	RHD2132 (32-channel amplifier)
Sampling rate, Hz	300	62, 125, 250, 500, 1000
Lithium-ion battery	LP301012 (30 mAh 3.7 V)	LP601120 (100 mAh 3.7 V)
Battery life, hours	≤3	>35
Size, mm (length × width × height)	20.31 × 13.39 × 7.95	18 × 13 × 3
Weight, grams (without battery)	1.79	0.95
Weight, grams (with battery)	2.31	3.19

**Table 3:** The comparison of the microprocessors in the WES 1.0 and 2.0 wireless modules.

Parameters	WES 1.0	WES 2.0
Architecture	Arm Cortex-M0+	Arm Cortex-M4
Instruction set architecture	Armv6-M	Armv7-M
Digital signal processing (DSP) extension	No	Yes
Hardware divide	No	Yes
DMIPS/MHz	1.26	0.99
CoreMark^®^/MHz	3.54	2.46
Frequency, MHz	64	64
Interfaces	I2C, I2S, SPI, UART, USART	2-channel 12-bit ADC, SPI, UART, TWI and QDEC
Internal RAM size, KB	36	24
Memory size, KB	128	192
Memory type	Flash	Flash

**Table 4:** The comparison to selected electrophysiology systems.

	Gagnon-Turcotte G. et al. [11]	Gagnon-Turcotte G. et al. [12]	Bilodeau G. et al. [13]	Wright J. et al. [14]	Wang H. et al. [15]	Sporer M. et al. [16]	This Work
Year	2016–2017	2019	2021	2022	2022	2022	2023
Data transmission type	2.4 GHz	2.4 GHz	2.4 GHz	ESB	BLE	BLE	BLE
Receiver–transmitter	nRF24l01p	nRF24L01p	nRF24l01p	nRF52840	nRF52832	NRF52832	NRF52805
Wireless transmission rate, Mbit/s	1.4	1.4	1.4	2	1.4	1.4	1.4
Electrophysiology interface chip	RHD2132	Custom mixed-signal 0.13-μm CMOS system-on-chip	RHD2132	RHS2116	Dual op-amps full differential neural recording front-end structure	Analog front-end signal chain	RHD2132
Number of recording channels	32	10	32	8	2	32	32
Sampling rate per channel, kHz	20	20	20	20	20	1	0.062, 0.125, 0.25, 0.5, 1
Lithium-ion 3.7 V Battery, mAh	100	40	100	10	30	45	100
Battery life, hours	1.75	2.66	1.56	0.58	1.5	5.9	38
Power consumption	31.9 mW/channel, 3.72 mW/channel (without stimulation)	21 mW	37 mA	62 mW	1.5 mW/channel ~10 mW (entire system)	12.6 mW	2.5 mA (standby), 2.6 mA (data transfer)
Weight, gram (without battery)	2.8	2.2	1.7	2.8	0.257	1.5	0.95
Weight, gram (with battery)	4.9	3.0	4.68	3.3	0.955	3.2	3.19
Connectors	Molex SlimStack, 0537480208	NA	Molex SlimStack, 0559090574	NA	NA	Omnetics PZN	Hirose Electric, BM10NB(0.8)-20DS-0.4V(51)
Size, mm	17 × 18 × 10	–	28 × 15 × 11	19.9 × 18.1 × 6.6	9 × 7 × 5	20 × 23 × 9	18 × 13 × 3
Additional options							
Electrical neurostimulation	no	no	no	yes	no	no	no
Optrode connection	yes	yes	yes	no	no	no	yes
On-board LED	yes	yes	yes	no	no	no	no

ESB—Enhanced ShockBurst protocol. BLE—Bluetooth low energy.

## Data Availability

The data of this study are openly available at the following URL: https://github.com/lmn-projects/WES-2.0 (accessed on 1 September 2023).

## Appendix B

### Appendix B.1. Programming nRF Microcontrollers

The J-Flash Lite utility included in the Segger software package (Segger Microcontroller, Germany) is used for programming:

https://www.segger.com/downloads/jlink#J-LinkSoftwareAndDocumentationPack (accessed on 1 September 2023).

1. Start J-Flash Lite utility, select the appropriate controller model, and then proceed by clicking on the ‘OK’ button.

2. To program the device, choose the firmware file and then press the ‘Program Device’ button.

3. The flashing process of the wireless module has been completed.

### Appendix B.2. ble_mouse.py Script Instruction

1. Activate Bluetooth on the computer, then connect the battery to the wireless module. Subsequently, establish a connection with the device ‘Mouse_UART’.
2. Identify the MAC address of ‘Mouse_UART’ and specify it in the ‘ble_mouse.py’ file on the ‘address’ line. Example: address = ‘D5:57:15:07:1D:A6’.
3. Ensure that Python is installed on your operating system. If it is not already installed, please proceed with the installation.
4. Navigate to the directory housing the ble_mouse.py program using Windows Explorer.
5. In the line displaying the current path, type ‘cmd’ and press the ‘Enter’ button. This action will initiate the standard Windows console at its present location, opening in a separate window.
6. Install the ‘bleak’ and ‘keyboard’ modules using the command ‘pip install bleak keyboard’.
7. Enter the launch command in the console line as follows: *ble_mouse.py* [*frequency*] [*list of channels*],

where frequency refers to the sampling rate, with the valid values being 1000, 500, 250, 125, or 62, and channel list denotes the specific channel numbers from which data will be received. These values range from one to thirty-two and are separated by a space. Based on the selected sample rate, the following number of channels are expected:
  ▪ 1000 Hz—two channels;
  ▪ 500 Hz—four channels;
  ▪ 250 Hz—eight channels;
  ▪ 125 Hz—sixteen channels;
  ▪ 62 Hz—all thirty-two channels are read; channel numbers need not be specified.

8. Press the ‘Enter’ button to run the command. The script will run and save the received data into a .csv file.
9. To stop the script, press and hold the ‘Space’ button.

## References

[1] Davis K.E., Fox S., Gigg J. (2014). Increased hippocampal excitability in the 3xTgAD mouse model for Alzheimer’s disease in vivo. PLoS ONE.

[2] Bezprozvanny I. (2022). Alzheimer’s disease—Where do we go from here?. Biochem. Biophys. Res. Commun..

[3] Besnard S., Decressac M., Denise P. (2010). In-vivo deep brain recordings of intranigral grafted cells in a mouse model of Parkinson’s disease. Neuroreport.

[4] Donzis E.J., Holley S.M., Cepeda C., Levine M.S. (2018). Neurophysiological Assessment of Huntington’s Disease Model Mice. Methods Mol. Biol..

[5] Gill B.J.A., Wu X., Khan F.A., Sosunov A.A., Liou J.Y., Dovas A., Eissa T.L., Banu M.A., Bateman L.M., McKhann G.M. (2020). Ex vivo multi-electrode analysis reveals spatiotemporal dynamics of ictal behavior at the infiltrated margin of glioma. Neurobiol. Dis..

[6] Bruno G., Colistra N., Melle G., Cerea A., Hubarevich A., Deleye L., De Angelis F., Dipalo M. (2020). Microfluidic Multielectrode Arrays for Spatially Localized Drug Delivery and Electrical Recordings of Primary Neuronal Cultures. Front. Bioeng. Biotechnol..

[7] Jang J.W., Kang Y.N., Seo H.W., Kim B., Choe H.K., Park S.H., Lee M.G., Kim S. (2021). Long-termin-vivorecording performance of flexible penetrating microelectrode arrays. J. Neural Eng..

[8] Ghomashchi A., Zheng Z., Majaj N., Trumpis M., Kiorpes L., Viventi J. (2014). A low-cost, open-source, wireless electrophysiology system. Annu. Int. Conf. IEEE Eng. Med. Biol. Soc..

[9] Cheng N., Murari K. (2020). OSERR: An open-source standalone electrophysiology recording system for rodents. Sci. Rep..

[10] Erofeev A., Kazakov D., Makarevich N., Bolshakova A., Gerasimov E., Nekrasov A., Kazakin A., Komarevtsev I., Bolsunovskaja M., Bezprozvanny I. (2021). An Open-Source Wireless Electrophysiological Complex for In Vivo Recording Neuronal Activity in the Rodent’s Brain. Sensors.

[11] Gagnon-Turcotte G., LeChasseur Y., Bories C., Messaddeq Y., De Koninck Y., Gosselin B. (2017). A Wireless Headstage for Combined Optogenetics and Multichannel Electrophysiological Recording. IEEE Trans. Biomed. Circuits Syst..

[12] Gagnon-Turcotte G., Keramidis I., Ethier C., De Koninck Y., Gosselin B. (2019). A Wireless Electro-Optic Headstage With a 0.13-mum CMOS Custom Integrated DWT Neural Signal Decoder for Closed-Loop Optogenetics. IEEE Trans. Biomed. Circuits Syst..

[13] Bilodeau G., Gagnon-Turcotte G., Gagnon L.L., Keramidis I., Timofeev I., De Koninck Y., Ethier C., Gosselin B. (2021). A Wireless Electro-Optic Platform for Multimodal Electrophysiology and Optogenetics in Freely Moving Rodents. Front. Neurosci..

[14] Wright J.P., Mughrabi I.T., Wong J., Mathew J., Jayaprakash N., Crosfield C., Chang E.H., Chavan S.S., Tracey K.J., Pavlov V.A. (2022). A fully implantable wireless bidirectional neuromodulation system for mice. Biosens. Bioelectron..

[15] Wang H., Ma Q., Chen K., Zhang H., Yang Y., Zheng N., Hong H. (2022). An Ultra-Low-Noise, Low Power and Miniaturized Dual-Channel Wireless Neural Recording Microsystem. Biosensors.

[16] Sporer M., Reich S., Mandry H., Becker J., Ortmanns M. A Wireless Headstage Prototype Based on a Neurorecorder IC. Proceedings of the 2022 IEEE Biomedical Circuits and Systems Conference (BioCAS).

